# Characterization of two ultra-rare CFTR variants, P.Leu999del and P.Glu1104Lys, with unknown theratyping profiles

**DOI:** 10.1186/s13023-026-04415-1

**Published:** 2026-06-02

**Authors:** Tereza Dousova, Lucie Borek-Dohalska, Stepanka Novotna, Veronika Skalicka, Malgorzata Libik, Milan Macek, Pavel Drevinek

**Affiliations:** 1 Department of Pediatrics, Second Faculty of Medicine, Charles University and Motol and Homolka University Hospital, Prague, Czech Republic; 2 Department of Medical Microbiology, Second Faculty of Medicine, Charles University and Motol and Homolka University Hospital, Prague, Czech Republic; 3 Department of Biology and Medical Genetics, Second Faculty of Medicine, Charles University and Motol and Homolka University Hospital, Prague, Czech Republic

**Keywords:** Cystic fibrosis, CFTR variants, CFTR modulators, Theranostics, Intestinal organoids, Nasal epithelial cells

## Abstract

**Background:**

Individuals carrying ultra-rare CFTR variants remain untreated with CFTR modulator therapies due to a lack of functional and clinical data supporting variant-specific responsiveness. This study aimed to functionally characterize two ultra-rare CFTR variants, p.Glu1104Lys (E1104K) and p.Leu999del (L999del), each found *in trans* with the minimal function variant G542X, and to evaluate their responsiveness to the triple-combination regimens elexacaftor/tezacaftor/ivacaftor (ELX/TEZ/IVA) and vanzacaftor/tezacaftor/ivacaftor (VAN/TEZ/IVA).

**Results:**

Patient-derived intestinal organoids (PDIOs) of two people with cystic fibrosis (G542X/E1104K and G542X/L999del) were cultured from rectal biopsies and subjected to forskolin-induced swelling (FIS) assays. CFTR function was quantified by calculating the area under the curve after 60 minutes. Human nasal epithelial cells (HNECs) were obtained by nasal brushing, expanded under air–liquid interface conditions, and evaluated in Ussing chambers. CFTRinh-172-sensitive stimulated currents (ΔCFTRinh-172 I_SC_) were expressed as percentages of the mean response of healthy controls. Both PDIOs exhibited visible swelling under untreated conditions, indicating residual CFTR function. Following pretreatment with ELX/TEZ or VAN/TEZ, both showed marked pre-swelling limiting quantitative analysis to qualitative assessment. In HNECs, residual CFTR activity reached 15% and 13% of WT levels for E1104K and L999del, respectively. Upon modulator treatment, CFTR activity increased to 90% (ELX/TEZ) and 97% (VAN/TEZ) in E1104K, and to 46% and 56% in L999del.

**Conclusions:**

E1104K and L999del exhibit measurable residual CFTR function and in vitro responsiveness to two CFTRm triple-combination regimens. These findings support theranostic approaches to guide therapy in individuals with ultra-rare CFTR genotypes, while clinical use remains dependent on regulatory approval and individualized patient assessment.

**Supplementary Information:**

The online version contains supplementary material available at 10.1186/s13023-026-04415-1.

## Background

CFTR modulator (CFTRm) therapies have profoundly changed the treatment landscape of cystic fibrosis (CF), leading to significant improvements in clinical outcomes and quality of life [1]. The triple combination elexacaftor/tezacaftor/ivacaftor (ELX/TEZ/IVA) is currently approved for approximately 90% of people with CF (pwCF), mainly those carrying at least one copy of the p.Phe508del (F508del) pathogenic variant (previously called mutations), the most common class II variant in populations of European descent [2]. Its CFTRm eligibility is based on solid clinical evidence showing significant improvements in lung function, sweat chloride concentration (SCC), and health-related quality of life [3, 4]. Since 2020, the U.S. Food and Drug Administration (FDA) expanded eligibility to include 271 rare CFTR variants based on clinical and in vitro data generated in Fischer rat thyroid (FRT) cells [5]. In 2025, the European Medicines Agency (EMA) further broadened the indications to all individuals carrying at least one non–class I variant and, importantly, officially recognized patient-derived intestinal organoid (PDIO) data as sufficient functional evidence for establishing CFTR variant responsiveness [6, 7]. Recently, another “triple combination,” comprising vanzacaftor/tezacaftor/deutivacaftor (VAN/TEZ/D-IVA), was approved in the United States, followed by regulatory approval in the UK and European Union (EU) [8–10]. Its indications mostly mirror those of ELX/TEZ/IVA, with the addition of 31 rare variants supported by FRT cell–based in vitro data in the United States. Clinical trial data suggest that VAN/TEZ/D-IVA provides comparable improvements in lung function to ELX/TEZ/IVA but result in a more significant reduction in SCC, suggesting enhanced CFTR rescue [11].

Despite these advances, approximately 4–5% of pwCF harbour ultra-rare or previously unclassified *CFTR* variants and are not covered by current regulatory indications for CFTRm therapy [12]. Here, ineligibility refers to the absence of a given variant from FDA- or EMA-approved indication labels, rather than to absolute lack of treatment access. Both CFTR variants investigated in this study, p.Glu1104Lys (E1104K) and p.Leu999del (L999del), had not been functionally classified prior to this work and were therefore not included in existing FDA-approved variant-specific indications. Under EMA regulations, treatment eligibility in such cases depends on functional evidence demonstrating that the variant does not belong to class I. The p.Glu1104Lys (E1104K) is a missense variant located within membrane-spanning domain 2 (MSD2), a region critical for CFTR channel architecture and gating. Although it is present in CFTR1 database [13], it is absent in the public versions of CFTR2 [14] and CFTR France [15] databases. Its therapeutic responsiveness has only been assessed in vitro using FRT cell lines and found to be borderline responsive to ELX/TEZ/IVA CFTRm [16]. The other variant under investigation, the p.Leu999del (L999del), is a rare in-frame deletion in MSD2. This variant is also missing in public versions of both databases and to date, no functional, clinical, or in silico analyses have been published to clarify the impact of this mutation on CFTR processing, channel activity, or drug responsiveness. Given the increasing availability of CFTRm therapies, our study aimed to provide individualized in vitro characterization of these ultra-rare variants using PDIOs and HNECs. We evaluated responsiveness to the triple-combination regimens elexacaftor/tezacaftor/ivacaftor (ELX/TEZ/IVA) and vanzacaftor/tezacaftor/ivacaftor (VAN/TEZ/IVA). Although D-IVA is used in the clinically approved VAN/TEZ/D-IVA regimen, IVA was used in our in vitro experiments.

## Methods

### CF subjects, ethics approval, and analysis of the entire CFTR gene

The Motol University Hospital Ethics Committee approved this study ethically, and all study subjects and/or their legal representatives provided informed consent. Index case DNA was isolated from blood leukocytes. Initially, the 50 most common CFTR mutations in European-derived populations were identified using the ElucigeneTm CF-EU2 assay (Elucigene Diagnostics, United Kingdom), followed by extensive sequencing of the entire CFTR coding region, adjacent splice site junctions, selected CNVs, and several introns with a locus-specific library preparation assay (CFTR NGS assayTm; Devyser, Sweden; http://www.devyser.com) on the MiSeq SystemTm next-generation sequencing platform (Illumina, USA; http://www.Illumina.com). Bioinformatic analysis employed the comprehensive SOPHiA DDM Platform for Hereditary Disorders (http://www.sophiagenetics.com/technology/sophia-ddm-for-genomics). Additionally, multiplex ligation-dependent probe amplification (MLPA) analysis of intra-CFTR rearrangements and CNVs was performed using the SALSA MLPA P091 CFTR Assay, with raw data analyzed on the proprietary software Coffalyser.NetTm (MRC-Holland, The Netherlands; http://www.MRCholland.com) to supplement the analysis within the CFTR NGS assay.

### Rectal biopsies, intestinal organoids and forskolin-induced swelling (FIS) assay

Superficial rectal mucosa biopsies were collected and immediately placed in ice-cold medium. Crypt isolation, organoid culture, and the FIS assay were carried out as previously described [17]. In brief, PDIOs were mechanically dissociated and seeded into 96well plates. Organoids were either left untreated (pretreated with DMSO as a vehicle) or pretreated for approximately 24 hours with ELX/TEZ (2 μM/3 μM) or VAN/TEZ (0.2 μM/3 μM). Prior to the FIS assay, PDIOs were stained with 3 μM calcein green (Invitrogen, USA) for 30 minutes. The assay was initiated by adding solutions containing increasing concentrations of forskolin (Fsk). Untreated PDIOs were stimulated with Fsk alone, whereas PDIOs pretreated with CFTR correctors were stimulated with Fsk in the presence of 3 μM IVA. PDIOs swelling was monitored and recorded over the course of 1 hour using a confocal microscope (Leica TCS SP8, Leica Microsystems, Germany). Images were analyzed with CellProfiler software, and the area under the curve (AUC; *t* = 60 minutes; baseline = 100%) was calculated using GraphPad Prism 7.0. Organoids derived from a healthy control (wild type; WT) and a subject homozygous for the F508del variant served as references. Experiments for each individual patient were performed in three independent experimental runs. On each run, two technical replicates per condition were analyzed, including untreated, ELX/TEZ-pretreated, and VAN/TEZ-pretreated organoids, resulting in a total of six technical measurements per condition per patient across three independent runs.

### Nasal brushing, HNECs cultures, Ussing chamber measurements

HNECs from pwCF carrying G542X/E1104K (*n* = 1), G542X/L999del (*n* = 1), G542X/G542X (*n* = 1), F508del/F508 del (*n* = 8) genotypes and WT donors (*n* = 8) obtained by nasal brushing were handled as previously described [18]. Electrophysiological analyses were performed in an Ussing chamber (VCC MC6, Physiologic Instruments, San Diego, CA). HNECs were maintained under short-circuit conditions by clamping the transepithelial voltage at 0 mV. Prior to each experiment, the offset between the voltage electrodes and the fluid resistance was compensated. Recordings were performed with both the apical and basolateral chambers filled with 2.5 ml of defined solutions. The basolateral solution contained 145 mM NaCl, 3.3 mM K₂HPO₄, 10 mM HEPES, 10 mM D-glucose, 1.2 mM CaCl₂, and 1.2 mM MgCl₂ (pH 7.35). In the apical solution, NaCl was replaced by Na-gluconate. All solutions were equilibrated with 95% O₂/5% CO₂, and experiments were conducted at 37 °C. Short-circuit current (I_SC_) was measured after addition of specific compounds: 1) 100 μM amiloride (AMIL), 2) 10 μM Fsk and 100 μM IBMX, 3) 10 μM IVA (in pwCF only), 4) 10 μM CFTRinh-172 and finally 5) 100 μM ATP. The following parameter were calculated: 1) Δamiloride-insensitive I_SC_ - difference between I_SC_ after addition of amiloride and I_SC_ after addition of CFTRinh-172, 2) ΔFsk/IBMX(+IVA) I_SC_ - difference between I_SC_ after addition of Fsk/IBMX (with IVA in pwCF and without IVA in WT) and I_SC_ after addition of amiloride, 3) ΔCFTRinh-172 I_SC_ - difference between I_SC_ after addition of Fsk/IBMX (with IVA in pwCF and without IVA in WT) and I_SC_ after addition of CFTRinh-172. HNECs were left untreated or pretreated with 3 μM ELX/ 3 μM TEZ and 3 μM VAN/3 μM TEZ 48 hours before electrophysiological experiments. I_SC_ change after the addition of amiloride, stimulation with Fsk/IBMX/IVA (ΔFsk/IBMX+IVA I_SC_; modulator-induced CFTR activity) and its inhibition by CFTRinh-172 (ΔCFTRinh-172 I_SC_; CFTRinh-172-sensitive stimulated current) were calculated and normalized to WT values. Experiments were conducted in three separate Ussing chamber runs per patient. Within each run, technical replicates per condition (untreated, ELX/TEZ-pretreated and VAN/TEZ-pretreated HNECs) for each patient was measured. Additionally, experiments for each WT were performed in one or two separate Ussing chamber runs.

### Statistical analyses

For PDIOs experiments, FIS was quantified as AUC where applicable. Each condition was tested in six technical replicates. The mean ± standard deviation (SD) of all 6 replicates was calculated.

In HNEC experiments, CFTR genotypes (G542X/E1104K, G542X/L999del, and G542X/G542X), are based on single-donor evidence (*n* = 1). Using chamber analyses for these genotypes are reported as insert-level data points (3–4 technical replicates) and donor means ± SD (Supplementary Table 1). For F508del/F508del group (*n* = 8) and WT controls (*n* = 8), insert-level measurements (3 technical replicates for F508del/F508del and 3–7 for WT) were first averaged within each donor, and subsequently used to calculate group-level mean ± SD. These group-level values were used to define thresholds for F508del/F508del and WT CFTR activity (Supplementary Tables 2 and 3). WT CFTR activity was defined as the mean of ΔCFTRinh-172 I_SC_ across all 8 WT donors (Supplementary Table 2) and served as the reference for expressing CFTR function in HNECs carrying ultra-rare CFTR genotypes.

## Results

### Clinical characterization of pwCF carrying the E1104K or L999del variants

**Patient 1 (CF1)** is a 15-year-old female diagnosed with CF at 6 weeks of age through nationwide newborn screening (NBS), which showed an elevated immunoreactive trypsinogen (IRT) level of 87.8 ng/mL. Confirmatory testing indicated a sweat chloride concentration (SCC) of 75 mmol/L, and CFTR sequencing of the entire CFTR coding region identified compound heterozygosity for G542X and E1104K (p.Glu1104Lys; c.3310 G > A). She remained pancreatic sufficient (PS), with consistently normal fecal elastase (FE) levels (range, 507–2430 µg/g). Nutritional status also remains well-preserved, with a BMI of 22.4 (+2.4 standard deviation (SD)). Microbiological surveillance has demonstrated intermittent bronchial colonization with *Pseudomonas aeruginosa* and chronic infection with *Staphylococcus aureus.* Pulmonary function testing showed mild peripheral obstructive changes, with a predicted forced expiratory volume in one second (FEV₁) of 105%. However, the maximal expiratory flow at 25% of forced vital capacity (MEF₂₅) was reduced to 59% of the predicted value, indicating small airway obstruction. Additionally, high-resolution computed tomography (HRCT) revealed early bronchiectasis since the age of seven.

The E1104K (p.Glu1104Lys; c.3310 G > A) variant is not listed in the ClinVar database [19]. Bioinformatic analysis of the E1104K mutation shows it affects a conserved CFTR nucleotide with a CADD score of 27.8 [20] and a REVEL score of 0.877 [21]. The variant is classified as likely pathogenic based on the standard ACMG/AMP criteria [22], supported by the AlphaMissense score [23] of 0.9363. The variant is also absent in control chromosomes in the current GnomAD database [24]. Finally, another variant affecting the same amino acid position but resulting in a different change (e.g., E1104V) was also classified as „likely pathogenic.”

**Patient 2 (CF2**) is a 2-year-old boy diagnosed with CF at 6 weeks of age following NBS, which showed an IRT level of 91.8 ng/mL. Sweat chloride testing confirmed high chloride levels (62 mmol/L), and CFTR sequencing found compound heterozygosity for G542X and the very rare L999del variant. He still preserves pancreatic sufficiency, with FE levels ranging from 460 to 2810 µg/g. His nutritional status remains normal, with a BMI of 16.6 (− 0.1 SD). The patient has stayed asymptomatic, with no signs of chronic airway colonization. Pulmonary function testing has not been done yet due to his age.

The L999del (p.Leu999del; c.2991_2993del) variant results in an in-frame deletion of a single amino acid in the CFTR gene. The affected residue is located in an evolutionarily conserved region, as indicated by a high PhyloP100 conservation score (9.537). No splicing-specific analyses were performed in this study. Therefore, according to ACMG/AMP guidelines and CFTR-specific recommendations, this variant was classified as a variant of uncertain significance (VUS).

### Function of E1104K and L999del-CFTR in intestinal organoids and response to CFTR modulators

**Fig. 1:**
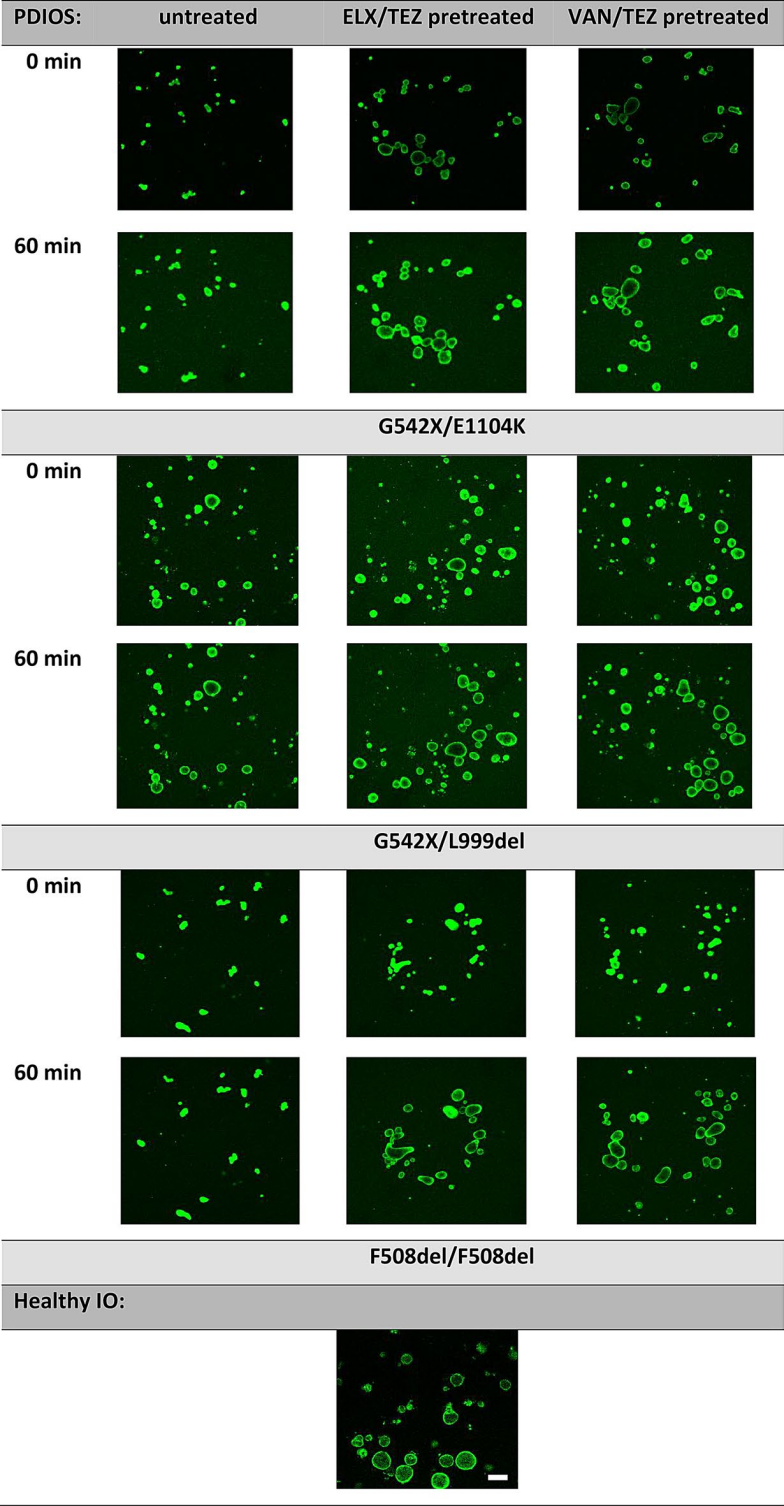
FIS assay in PDIOs. Representative images of calcein green-stained PDIOs were taken before (0 min) and after (60 min) stimulation with Fsk (0.128 µM). Untreated PDIOs were stimulated with Fsk alone, whereas ELX/TEZ- and VAN/TEZ-pretreated PDIOs were stimulated with Fsk in the presence of ivacaftor (IVA, 3 µM). PDIOs from pwCF carrying G542X/E1104K, G542X/L999del, and F508del/F508del variants were either a) untreated, b) pre-treated with ELX (2 µM)/TEZ (3 µM), or c) VAN (0.2 µM)/TEZ (3 µM) 24 hours before the FIS experiment. A representative image of calcein green-stained organoids from healthy controls is also shown (scale bar = 200 µm)

We analyzed PDIOs from individuals carrying the G542X/E1104K and G542X/L999del genotypes. Under untreated conditions at baseline (time 0), PDIOs from both individuals displayed varying degrees of pre-swelling. Pre-swelling was more pronounced in G542X/L999del PDIOs, which showed luminal morphology and size closely resembling those of WT, and clearly exceeding the reduced luminal areas observed in F508del/F508del organoids. PDIOs from the G542X/E1104K individual demonstrated smaller luminal structures and distinct morphology. A comparative overview of baseline morphologies across all genotypes, including WT, is shown in Fig. 1.

**Fig. 2:**
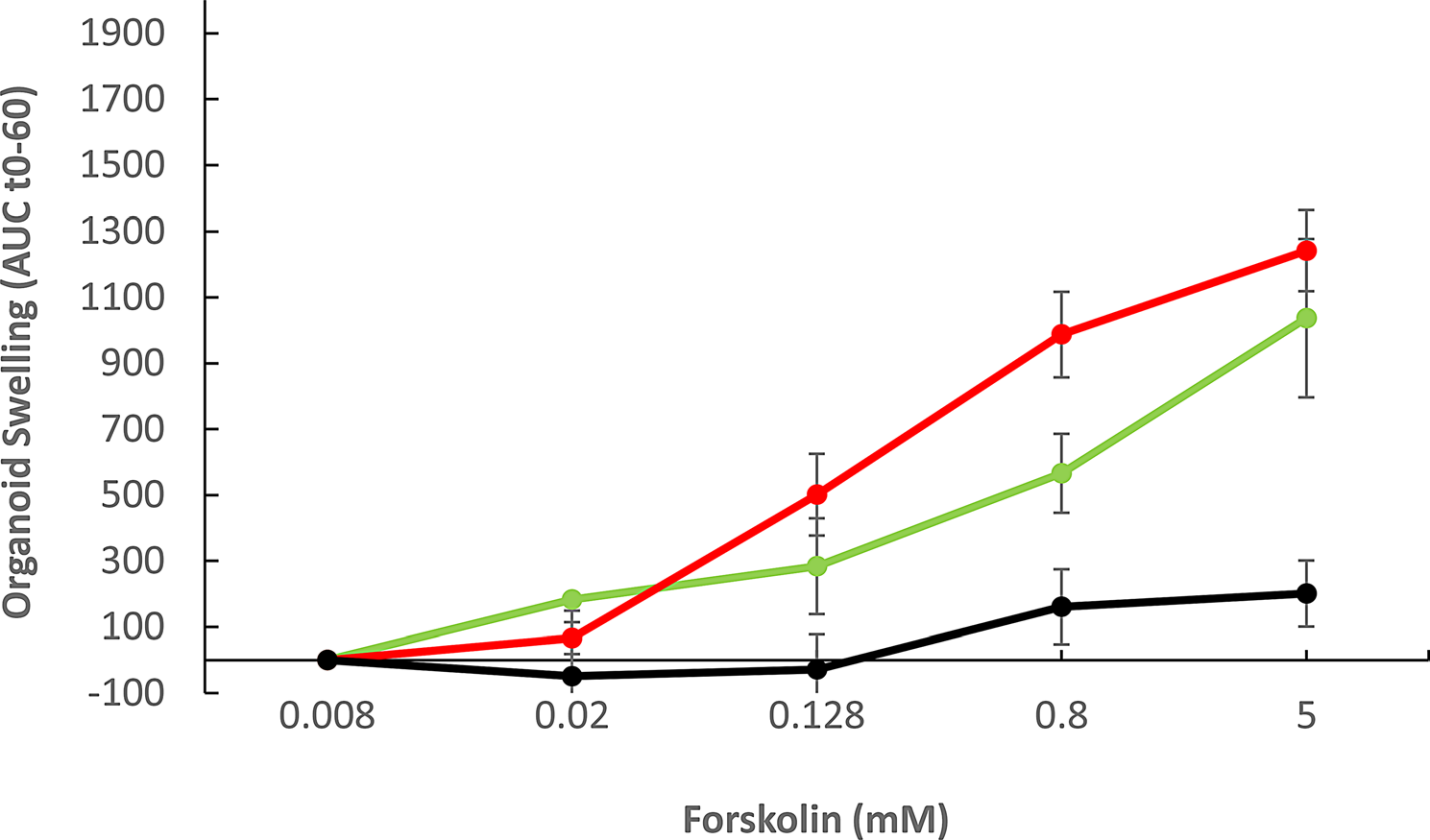
FIS assays and average AUC after 1-hour in PDIOs from pwCF carrying G542X/E1104K (green line), G542X/L999del (red line), F508del/F508del (black line). PDIOs were untreated and stimulated with different concentrations of Fsk. The error bars represent +/- 1 SD. Each experimental condition was performed in 6 technical replicates

Following only Fsk stimulation in the absence of ELX/TEZ or VAN/TEZ pretreatment, organoids from both individuals exhibited visible swelling over the 60-minute FIS assay, indicating preserved residual CFTR activity (Fig. 2). After 24-hour pretreatment with either ELX/TEZ or VAN/TEZ, organoids from both genotypes exhibited marked pre-swelling prior to Fsk and IVA stimulation, which precluded standard AUC-based quantification and allowed only qualitative assessment of the swelling response. Therefore, quantitative AUC data are not shown, whereas representative qualitative images are presented (Fig. 1). In contrast, F508del/F508del organoids demonstrated robust modulator-induced swelling, consistent with their known CFTRm responsiveness (Fig. 1).

### Function of E1104K and L999del-CFTR in Ussing chamber and response to CFTRm

**Fig. 3:**
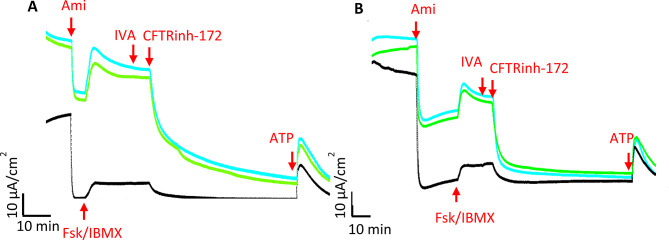
Representative tracings of I_SC_ measurements in HNECs from pwCF carrying G542X/E1104K (**A**) and G542X/L999del (**B**) genotypes, untreated (black) and pretreated with ELX/TEZ (green) and VAN/TEZ (blue). I_SC_ was measured after addition of 100 μM amiloride (Ami); 100 μM 3-isobutyl-1-methylxanthine (IBMX) and 10 μM forskolin (Fsk), followed by addition of 10 μM ivacaftor (IVA), 10 μM CFTRinh-172 and 100 μM ATP

**Fig. 4:**
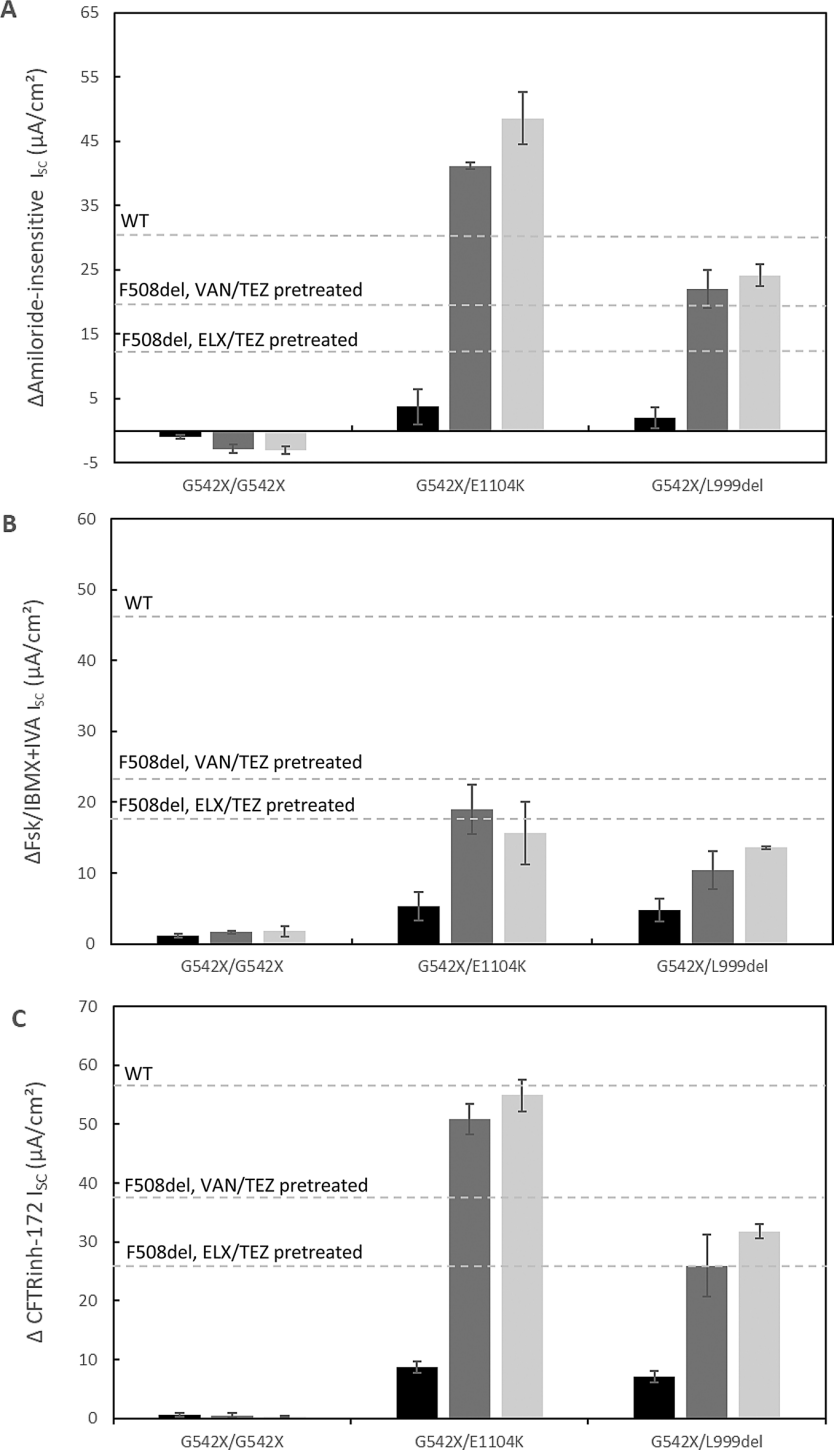
I_SC_ differences in HNECs from pwCF with, G542X/G542X (*n* = 1), G542X/E1104K (*n* = 1), G542X/L999del (*n* = 1). Δ Amiloride-insensitive I_SC_ (**A**) - difference between I_SC_ after addition of amiloride and I_SC_ after addition of CFTRinh-172; ΔFsk/IBMX+IVA I_SC_ (**B**) - difference between I_SC_ after addition of Fsk/IBMX and IVA and I_SC_ after addition of amiloride; ΔCFTRinh-172 I_SC_ (**C**)- difference between I_SC_ after addition of Fsk/IBMX and IVA and I_SC_ after addition of CFTRinh-172. The experimental conditions ´untreated´ are in black, ´ELX/TEZ-pretreated´ in dark grey and ´VAN/TEZ-pretreated’ in light grey. Dashed line indicates reference threshold derived from WT (*n* = 8, Supplementary Table 2) and F508del/F508del donors (*n* = 8, Supplementary Table 3)

Representative I_SC_ tracings of HNECs derived from pwCF carrying G542X/E1104K and G542X/L999del genotypes, either untreated or pretreated with ELX/TEZ or VAN/TEZ are shown in Fig. 3. The findings are presented as n-of-1 functional phenotyping because each ultra-rare genotype was represented by a single donor. Both E1104K and L999del variants exhibited measurable residual CFTR activity. Based on CFTRinh-172-sensitive stimulated current, residual CFTR function reached 15% of WT levels in G542X/E1104K HNECs and 13% in G542X/L999del HNECs. Following pre-treatment with either ELX/TEZ or VAN/TEZ and acute IVA stimulation, a marked increase in CFTR activity was observed in both genotypes. In the G542X/E1104K donor, CFTR function increased to 90% of WT levels following ELX/TEZ and to 97% of WT following VAN/TEZ. In the G542X/L999del donor, CFTR activity increased to 46% and 56% of WT levels following ELX/TEZ and VAN/TEZ pretreatment. In the G542X/L999del donor, CFTR activity increased to 46% and 56% of WT levels following ELX/TEZ and VAN/TEZ pretreatment, respectively. In both cases, ΔCFTRinh-172 ISC values after pretreatment were within or above the reference range observed in F508del/F508del HNECs treated with ELX/TEZ or VAN/TEZ (Fig. 4C). As controls, HNECs from pwCF homozygous for G542X were used. No appreciable residual CFTR activity was detected in these cells and as expected, pretreatment with ELX/TEZ or VAN/TEZ did not affect Δamiloride-insensitive I_SC_, ΔFsk/IBMX+IVA I_SC_ and ΔCFTRinh-172 I_SC_ (Fig. 4).

## Discussion

This study provides the first ex-vivo functional characterization of two ultra-rare CFTR variants, E1104K and L999del, each identified in *trans* with the minimal function G542X variant in two Czech pwCF. Using PDIOs and HNECs, we assessed CFTR activity and evaluated functional rescue following treatment with two CFTRm triple-combination regimens: ELX/TEZ/IVA and VAN/TEZ/IVA. Although deutivacaftor (D-IVA) is used in the clinically approved VAN/TEZ/D-IVA regimen, ivacaftor (IVA) was used in all in vitro experiments, as pharmacokinetic differences between these potentiators are not relevant under experimental conditions and do not materially affect CFTR potentiation.

In the FIS assay performed on PDIOs, we observed a marked degree of residual CFTR function in both cases, as evidenced by a significantly visible lumen even before modulator treatment. Upon pretreatment with both CFTR modulator combinations (ELX/TEZ and VAN/TEZ), PDIOs were preswollen. This so-called “pre-swelling” may introduce mechanical constraints that limit further expansion of the organoid lumen, thereby underestimating the modulator-induced increase in CFTR activity. As a result, it becomes challenging—or even impossible—to accurately evaluate the standard FIS readout, the AUC. This limitation has been previously reported by other groups studying modulator responses in PDIOs [25, 26]. A more appropriate parameter in such cases is the steady-state lumen area (SLA), which indicates the baseline size of the organoid lumen before Fsk or CFTRm stimulation. SLA reflects the volume of fluid accumulated under unstimulated conditions and thus serves as an indirect indicator of residual CFTR function [27, 28]. Kroes et al. [29] recently showed the clinical relevance of SLA, establishing a threshold to distinguish responders among a panel of rare CFTR genotypes. Due to technical and analytical limitations, SLA measurements could not be performed in this study, and swelling after pretreatment was therefore assessed qualitatively only.

To circumvent the limitations of the FIS assay, we validated our findings using Ussing chamber measurements on matched HNECs from the same individuals. This approach has demonstrated robust predictive capacity for rare and ultra-rare variants [30, 31] and a common indicator of CFTR function, namely ΔI_SC_ CFTRinh-172 (expressed as percentage of WT activity), was used. First, we have determined the residual CFTR activity based on CFTRinh-172–sensitive stimulated current, which was for the L999del variant 13% and for the E1104K variant 15% of WT levels. These findings confirmed measurable residual function in both variants.

Following pretreatment with ELX/TEZ and VAN/TEZ, a marked enhancement of CFTR activity was observed in both genotypes. In G542X/E1104K HNECs, CFTR function increased to 90% of WT levels with ELX/TEZ/IVA and to 97% of WT levels with VAN/TEZ/IVA. In G542X/L999del HNECs, activity rose to 46% and 56% of WT levels with ELX/TEZ/IVA and VAN/TEZ/IVA, respectively. Although values were numerically higher for VAN/TEZ pretreatment, no formal comparative analysis was performed, and these findings should be interpreted descriptively. Nevertheless, these observations highlight robust in vitro responsiveness to CFTR modulation, irrespective of the specific triple-combination regimen administered.

Interestingly, E1104K was previously analyzed in FRT cells by Bihler et al. [16] and showed only a borderline response to ELX/TEZ/IVA in vitro treatment. The higher degree of CFTR activity and modulator responsiveness detected in patient-derived models in the present study likely reflects differences between heterologous expression systems and native epithelial cells. In particular, patient-derived models preserve the endogenous cellular context of CFTR regulation, which may influence the apparent magnitude of residual function and therapeutic response, as also suggested by recent studies supporting the use of theranostic approaches for rare CFTR variants [31, 32].

These data highlight the clinical importance of theranostic approaches that combine patient-derived functional testing with therapeutic decision-making. Unlike theratyping, which relies on heterologous expression systems, theranostics captures the behavior of individual variants in native tissues. Recently, EMA acknowledged that FIS assay data are adequate for modulator label extension decisions [33]. In both cases described here, our data support consideration of modulator therapy despite current genotype-based exclusion from approved indications.

From a clinical perspective, the present findings provide functional evidence that may inform individualized consideration of CFTR modulator therapy in both study individuals, rather than serving as a direct indication for treatment initiation. Both patients showed features of a mild CF phenotype, including pancreatic sufficiency and good nutritional status. Their SCCs—62 mmol/L in the L999del case and 75 mmol/L in the E1104K case—fall within the diagnostic range for CF yet are close to the lower diagnostic threshold, a range associated in unclassified CFTR variants with milder disease expression [34].

The clinical relevance of in vitro responsiveness differs between the two cases. The toddler carrying the L999del/G542X genotype remains asymptomatic, clinically stable, and without evidence of chronic airway colonization. In this context, preserved residual CFTR function may be sufficient to sustain a benign clinical course, rendering the decision to initiate CFTRm therapy particularly nuanced. In very young, asymptomatic patients, in vitro functional responsiveness alone may not justify immediate treatment initiation, especially given the limited data on long-term safety, potential off-target effects, and neuropsychiatric adverse events reported in this age group [35]. Moreover, while L999del variant demonstrated in vitro responsiveness to CFTR modulators, such findings do not establish pathogenicity under ACMG/AMP criteria, the variant therefore remains classified as a VUS and clinical interpretation must remain cautious. In contrast, the adolescent patient carrying the E1104K variant already demonstrates early pulmonary involvement, including mild airflow obstruction and radiological evidence of bronchiectasis. In this setting, in vitro evidence of substantial CFTR rescue provides a stronger clinical rationale for considering CFTRm therapy as part of an individualized treatment strategy.

Importantly, the initiation of CFTRm therapy may be influenced not only by clinical considerations and regulatory eligibility, but also by local access and reimbursement frameworks. In some healthcare systems, including our own, treatment approval is currently straightforward for individuals carrying at least one F508del allele, whereas patients with rare or previously unclassified CFTR variants may face additional barriers to treatment initiation, even when functional evidence supports potential benefit. This highlights the need to integrate patient-derived functional data into clinical and regulatory discussions, particularly for individuals with rare genotypes.

This study has several limitations. First, functional analyses of ultra-rare CFTR genotypes were based on single donors. While insert-level technical replicates provide within-donor measurements, conclusions for these genotypes represent n-of-1 evidence and require independent replication when additional donors become available. Second, although D-IVA is used as the potentiator in the clinically approved VAN/TEZ/D-IVA combination, IVA was used in all in vitro experiments. D-IVA differs from IVA primarily in its pharmacokinetic properties, enabling more stable systemic exposure and once-daily dosing in vivo. As these pharmacokinetic differences are not relevant in in vitro experimental systems, substitution of D-IVA with IVA is unlikely to have materially affected CFTR potentiation in our functional assays. Third, organoid-based assessment using the FIS assay was limited by baseline pre-swelling which precluded accurate modulator response quantification via AUC. Although Ussing chamber measurements in HNECs provided confirming data, retrospective SLA analysis, which could have enabled more precise basal CFTR assessment, was not feasible. Incorporation of SLA into future studies may allow more refined quantification of basal CFTR function and modulator responsiveness in PDIOs. Fourth, although both CFTRm combinations were tested in vitro, there was no available clinical data (e.g., SCC, lung function, and symptom progression) to evaluate real-world treatment responses. And finally, regulatory and reimbursement restrictions may limit the clinical use of functionally guided treatment strategies for patients with „officially“unapproved CFTR genotypes.

## Conclusion

Both E1104K and L999del showed measurable residual CFTR activity and clear in vitro responsiveness to ELX/TEZ/IVA and VAN/TEZ/IVA. These findings support the use of individualized theranostic approaches to inform clinical decision-making, while cautious interpretation and clinical validation remain essential.

## Electronic supplementary material

Below is the link to the electronic supplementary material.

Supplementary material 1

Supplementary material 2

Supplementary material 3

## Data Availability

The datasets used and analysed during the current study are available from the corresponding author on reasonable request.

## Abbreviations

ALI: Air–liquid interface
AUC: Area under the curve
CF: Cystic fibrosis
CFTR: Cystic fibrosis transmembrane conductance regulator
CFTRinh-172: CFTR inhibitor 172
ELX: Elexacaftor
FIS: Forskolin-induced swelling
HNEC: Human nasal epithelial cell
I_SC_: Short-circuit current
IVA: Ivacaftor
MSD2: Membrane-spanning domain 2
PDIO: Patient-derived intestinal organoid
SCC: Sweat chloride concentration
TEZ: Tezacaftor
VAN: Vanzacaftor
WT: Wild type
