# Supplementary material for: Characterization of two ultra-rare CFTR variants, P.Leu999del and P.Glu1104Lys, with unknown theratyping profiles
Source: Orphanet J Rare Dis. 2026 Jun 2;21:270. doi: 10.1186/s13023-026-04415-1 (PMC13455205; doi:10.1186/s13023-026-04415-1)
Supplement: Supplementary file 1 [file 13023_2026_4415_MOESM1_ESM.docx]

**Supplementary Table 1.** **Insert-level CFTR functional measurements in human nasal epithelial cells (HNECs) derived from individuals with cystic fibrosis carrying ultra-rare CFTR genotypes (G542X/E1104K, and G542X/L999del) and G542X/G542X**, assessed under untreated conditions and after pretreatment with ELX/TEZ or VAN/TEZ followed by acute ivacaftor stimulation. For each genotype, all data originate from a single donor (n = 1); therefore, values are presented descriptively as insert-level measurements with corresponding donor means and standard deviations. ΔI_SC_ is expressed as Δamiloride-insensitive I_SC_, ΔFsk/IBMX+IVA I_SC_, and ΔCFTRinh-172 I_SC_ (µA/cm²).

| **Untreated HNECs** | **Insert** | **Δamiloride-insensitive I_SC_** | **ΔFsk/IBMX+IVA I_SC_** | **Δ CFTRinh - 172 I_SC_** |
| --- | --- | --- | --- | --- |
|  |  | (µA/cm^2^) | (µA/cm^2^) | (µA/cm^2^) |
| **G542X/G542X** | 1 | -0.80 | 1.00 | 0.80 |
|  | 2 | -1.20 | 1.30 | 0.40 |
|  | 3 | -1.00 | 1.15 | 0.60 |
|  | **Mean ± SD** | **-1,00** ± **0.28** | **1.15** ± **0.21** | **0.60** ± **0.28** |
| **G542X/E1104K** | 1 | 0.50 | 7.50 | 7.80 |
|  | 2 | 5.20 | 4.90 | 9.73 |
|  | 3 | 5.50 | 3.49 | 8.50 |
|  | **Mean ± SD** | **3.73** ± **2.80** | **5.30** ± **2.03** | **8.68** ± **0.98** |
| **G542X/L999del** | 1 | 0.30 | 5.09 | 7.22 |
|  | 2 | 3.80 | 4.20 | 6.00 |
|  | 3 | 0.40 | 6.90 | 8.20 |
|  | 4 | 3.50 | 3.06 | 7.00 |
|  | **Mean ± SD** | **2.00** ± **1.65** | **4.81** ± **1.62** | **7.11** ± **0.90** |
| **ELX/TEZ-pretreated HNECs** |  |  |  |  |
| **G542X/G542X** | 1 | -2.30 | 1.50 | 0.80 |
|  | 2 | -3.30 | 1.80 | 0.20 |
|  | 3 | -2,80 | 1.65 | 0.50 |
|  | **Mean ± SD** | **-2.80** ± **0.71** | **1.65** ± **0.21** | **0.50** ± **0.42** |
| **G542X/E1104K** | 1 | 40.80 | 15.5 | 51.60 |
|  | 2 | 41.69 | 19.1 | 53.09 |
|  | 3 | 41.00 | 22.4 | 48.00 |
|  | **Mean ± SD** | **41.16** ± **0.47** | **19.00** ± **3.45** | **50.90** ± **2.62** |
| **G542X/L999del** | 1 | 19.26 | 7.04 | 21.01 |
|  | 2 | 25.13 | 10.40 | 27.30 |
|  | 3 | 19.56 | 10.54 | 22.90 |
|  | 4 | 24.06 | 13.70 | 32.72 |
|  | **Mean ± SD** | **22.00** ± **3.03** | **10.42** ± **2.72** | **25.98** ± **5.21** |
| **VAN/TEZ-pretreated HNECs** |  |  |  |  |
| **G542X/G542X** | 1 | -3.60 | 2.50 | 0.40 |
|  | 2 | -2.90 | 1.80 | 0.30 |
|  | 3 | -2.40 | 1.00 | 0.40 |
|  | **Mean ± SD** | **-2.97** ± **0.60** | **1.77** ± **0.75** | **0.37** ± **0.06** |
| **G542X/E1104K** | 1 | 48.30 | 18.00 | 56.80 |
|  | 2 | 52.80 | 10.60 | 55.90 |
|  | 3 | 44.61 | 18.30 | 51.81 |
|  | **Mean ± SD** | **48.57** ± **4.10** | **15.63** ± **4.36** | **54.84** ± **2.66** |
| **G542X/L999del** | 1 | 22.47 | 13.80 | 30.42 |
|  | 2 | 23.87 | 13.40 | 31.54 |
|  | 3 | 25.91 | 13.60 | 33.31 |
|  | 4 | 24.30 | 13.50 | 32.00 |
|  | **Mean ± SD** | **24.14** ± **1.42** | **13.58** ± **0.17** | **31.82** ± **1.20** |
