# Supplementary material for: Characterization of two ultra-rare CFTR variants, P.Leu999del and P.Glu1104Lys, with unknown theratyping profiles
Source: Orphanet J Rare Dis. 2026 Jun 2;21:270. doi: 10.1186/s13023-026-04415-1 (PMC13455205; doi:10.1186/s13023-026-04415-1)
Supplement: Supplementary file 2 [file 13023_2026_4415_MOESM2_ESM.docx]

**Supplementary Table 2.** **CFTR functional parameters measured in human nasal epithelial cells (HNECs) derived from eight wild-type (WT) donors**. WT donor level are present as mean of 3-7 technical replicates. Group-level WT values were calculated as the mean ± SD of donor means. CFTR function is expressed as Δamiloride-insensitive I_SC_, ΔFsk/IBMX I_SC_, and ΔCFTRinh-172 I_SC_ (all in µA/cm²).

| **Donor** | **Δamiloride-insensitive I_SC_** | **ΔFsk/IBMX I_SC_** | **Δ CFTRinh-172 I_SC_** |
| --- | --- | --- | --- |
|  | (µA/cm²) | (µA/cm²) | (µA/cm²) |
| **WT1** | 39.68 | 48.19 | 58.02 |
| **WT2** | 26.77 | 32.87 | 67.14 |
| **WT3** | 38.43 | 50.18 | 54.85 |
| **WT4** | 23.64 | 51.10 | 59.10 |
| **WT5** | 41.91 | 89.80 | 70.97 |
| **WT6** | 21.50 | 45.70 | 63.16 |
| **WT7** | 23.67 | 32.87 | 40.94 |
| **WT8** | 29.86 | 20.04 | 36.86 |
| **WT-group** | **30.68 ± 7.63** | **46.34 ± 19.32** | **56.38 ± 11.22** |
|  |  |  |  |
