# Supplementary material for: Characterization of two ultra-rare CFTR variants, P.Leu999del and P.Glu1104Lys, with unknown theratyping profiles
Source: Orphanet J Rare Dis. 2026 Jun 2;21:270. doi: 10.1186/s13023-026-04415-1 (PMC13455205; doi:10.1186/s13023-026-04415-1)
Supplement: Supplementary file 3 [file 13023_2026_4415_MOESM3_ESM.docx]

| **ELX/TEZ-pretreated**  **HNECs** | **Δamiloride-insensitive I_SC_** | **ΔFsk/IBMX+IVA I_SC_** | **Δ CFTRinh-172 I_SC_** |
| --- | --- | --- | --- |
| **Donor ID** | (µA/cm²) | (µA/cm²) | (µA/cm²) |
| **ID1** | 6.93 | 20.05 | 24.02 |
| **ID2** | 4.93 | 23.21 | 27.03 |
| **ID3** | 28.73 | 20.84 | 31.05 |
| **ID4** | 1.54 | 6.78 | 6.89 |
| **ID5** | 22.23 | 23.59 | 38.4 |
| **ID6** | 17.60 | 27.22 | 43.47 |
| **ID7** | 7.94 | 7.75 | 14.80 |
| **ID8** | 11.27 | 11.81 | 19.30 |
| **Mean±SD** | **12.64±8.76** | **17.66±7.28** | **25.62±11.31** |
| **VAN/TEZ-pretreated**  **HNECs** | **Δamiloride-insensitive I_SC_** | **ΔFsk/IBMX+IVA I_SC_** | **Δ CFTR-inh 172 I_SC_** |
| **Donor ID** | (µA/cm²) | (µA/cm²) | (µA/cm²) |
| **ID1** | 14.34 | 34.81 | 47.05 |
| **ID2** | 16.10 | 30.18 | 43.98 |
| **ID3** | 27.25 | 23.18 | 37.82 |
| **ID4** | 7.76 | 8.59 | 14.73 |
| **ID5** | 33.70 | 25.55 | 47.85 |
| **ID6** | 34.73 | 31.93 | 62.85 |
| **ID7** | 8.84 | 13.62 | 21.48 |
| **ID8** | 13.30 | 17.12 | 25.94 |
| **Mean±SD** | **19.50±10.13** | **23.12±8.71** | **37.71±14.96** |

**Supplementary Table 3.** **CFTR functional parameters measured in human nasal epithelial cells (HNECs) derived from eight F508del/F508del pwCF,** assessed under pretreatment with ELX/TEZ or VAN/TEZ followed by acute ivacaftor stimulation. PwCF donor level are present as mean of 3 technical replicates. Group-level values were calculated as the mean ± SD of donor means. CFTR function is expressed as Δamiloride-insensitive I_SC_, ΔFsk/IBMX+IVA I_SC_, and ΔCFTRinh-172 I_SC_ (all in µA/cm²).
